# Comparative analysis of factors and barriers intervening in research participation among romanian and international medical graduates from one romanian medical faculty across three generations

**DOI:** 10.1186/s12909-024-05939-5

**Published:** 2024-09-19

**Authors:** Andreea Iulia Pop, Lucia Maria Lotrean

**Affiliations:** 1 Department of Community Medicine, Research Center in Preventive Medicine, Health Promotion and Sustainable Development, Iuliu Hatieganu University of Medicine and Pharmacy, Cluj-Napoca, 400012 Romania; 2 Department of Community Medicine, Research Center in Preventive Medicine, Health Promotion and Sustainable, Development Iuliu Hatieganu University of Medicine and Pharmacy, Cluj-Napoca, 400012 Romania

**Keywords:** Research participation, Medical graduates, Factors, Barriers, International medical education

## Abstract

**Objectives:**

This study focuses on the factors that encouraged engagement in research activities, as well as the barriers that restricted their involvement, until the final year of study at Iuliu Hatieganu University of Medicine and Pharmacy Cluj-Napoca, Faculty of Medicine. The main objectives of this study are to investigate potential disparities in research culture and student engagement in various research opportunities between Romanian and international medical graduates, as well as to conduct an examination of the observed patterns across various graduating years (2021–2023).

**Materials and methods:**

A cross-sectional investigation was conducted among graduate students of the Faculty of Medicine at the Iuliu Hațieganu University of Medicine and Pharmacy in Cluj-Napoca, Romania. From 2021 to 2023, all graduate students from the Romanian and international programs of the faculty were asked to participate in the study by filling out an anonymous online questionnaire. The final sample included 572 participants, of whom 392 were students from the Romanian section and 180 were students from international programs.

**Results:**

Motivation and personal interest drive research engagement, according to over half of graduates. For over one-third of graduates, institutional elements like financial support and education also play a major role, as does the desire to enhance their curriculum vitae. More than 25% of graduates value community influence, 70% of graduates attended medical congresses, 12–15% presented papers at medical conferences, 23% wrote medical articles, 10–15% published at least one scientific paper in medical journals, and 20% participated in medical school research projects. Comparative analysis showed that Romanian students start research earlier, attend more medical conferences, present posters, collect data for studies, and are more interested in publishing graduation thesis data in scientific journals. To encourage international students to participate in research, the study found that colleagues’ examples were more important, and both time and funds were key barriers. The research also shows that 2022 and 2023 graduates will organize more scientific conferences. According to the study, 2022 graduates began their research earlier than others.

**Conclusions:**

To increase student engagement in research activities, medical schools should prioritize the promotion of positive factors, minimize common barriers, offer customized support and resources, encourage collaborative research activities, and facilitate cross-cultural learning.

## Introduction

Medical schools play a crucial role in providing professionals with the necessary knowledge and skills to excel in their careers and contribute to the healthcare system [1]. The conventional medical education structure has created skilled and scientifically grounded healthcare professionals, but it is essential to adapt learning methods to align with new technological advances, diagnostic strategies, and medical treatments [2–4]. As healthcare environments change, medical education must advance to meet the evolving needs of patients and healthcare professionals. To stay informed about medical innovations, medical students must develop practical skills, synthesize information, and analyze vast amounts of information. They should also maximize interprofessional learning possibilities and balance the risks and benefits of various treatment options to provide the best possible patient care [5–7]. Currently, the requirement for enhanced competence in evidence-based medicine and concerns regarding the declining representation of physician-scientists have emphasized the necessity of promoting and encouraging research in medical education [8–11].

Research involves data collection and analysis, gathering key information, and then analyzing and interpreting that information according to academic and professional procedures. This suggests that research helps students develop critical thinking and problem-solving skills, which are crucial for healthcare practitioners, and it is essential to actively involve and motivate the upcoming generation of physician-scientists from earlier stages [12, 13]. Throughout the years, medical students have produced important innovations that have had a significant influence on current medicine through the adoption of evidence-based practice. Students made notable progress in several areas, such as the discovery of heparin, Raynaud’s disease, brachial plexus palsy, the atrioventricular node, ether anesthesia, penicillin, and insulin. Those historical examples play a crucial role in sustaining students’ motivation and developing their enthusiasm for excellence [14].

Scholarly research training programs help undergraduate medical students critically assess new information, communicate, and share research findings, making valuable contributions to the advancement of medical knowledge [15]. According to Yin et al., medical schools must prioritize research by offering enough opportunity, motivation, and assistance for student engagement [16]. Previous studies have investigated the training and participation of medical students in curricular and extracurricular research activities. Since the 1960s, some medical schools, such as Duke University and Stanford University, have offered research programs that accompany traditional education, widening students’ scientific knowledge and recruiting them to academic medicine [17]. Many medical schools nowadays offer students either mandatory or optional research alternatives that enhance their research skills. The Bologna process contributed to a restructuring of the medical undergraduate degree in Europe. It was launched in 1999 by several European countries with the goal of improving the acceptance and quality of higher education qualifications in the region. According to the Bologna process, European universities must evaluate scientific training and include research in their undergraduate medical degrees. As a result, medical students must complete a research project in order to graduate [8, 18]. To promote supervised research, Asian universities have implemented graduation requirements, which generally require undergraduate participation for a semester or academic year, either individually or with the support of the government [19]. The Liaison Committee on Medical Education (LCME) conducted a survey among 147 medical schools in the United States between 2017 and 2018, which revealed that 65 of them mandated medical students to conduct research [20]. On the other hand, extracurricular research programs (ERPs), such as summer research programs, Honours programs, or any other student research organizations worldwide, such as Harvard College Undergraduate Research Association, Cambridge University Students’ Clinical Research Society, and John B. Graham Medical Student Research Society, have been set up by many medical schools to encourage students to do research, develop an academic mindset, and become future doctors who are also scientists [21, 22].

Although the level to which medical graduates participate in research activities is influenced by a variety of factors and obstacles. Prior research has identified that to encourage and sustain the engagement of medical students in research, it is imperative to identify the fundamental factors that motivate their research efforts throughout the early years of their medical education [23]. In their study, Ommering et al. investigate the motivation of medical students to conduct research, and their findings suggest that students may have both intrinsic and extrinsic motivations. For extrinsic motivations, medical students may engage in research to enhance their training and career opportunities, such as securing a competitive residency. Furthermore, there is proof that students can be really interested in research and contribute out of satisfaction, as regards intrinsic motivations. Self-efficacy, curiosity, and challenge, prior training in scientific research, supportive teachers, and an environment that encourages research are the valuable motivational variables [23, 24]. While there is a tendency to refine involvement in research during medical school, the literature highlights both institutional and non-institutional barriers to successful participation. Previous studies have found several common barriers to research involvement, such as time constraints, insufficient funds, insufficient support from mentors, and a lack of knowledge and experience. Thus, Andrea and Sarah Cuschieri found that medical graduates often receive inadequate assistance and direction from faculty members and mentors, insufficient resources for carrying out research, minimal opportunities to participate in scientific initiatives, and a lack of motivation [25]. Griffin and Hindocha also highlighted barriers perceived by medical students to publishing, such as a lack of opportunities to conduct research, insufficient support from seniors, limited education on writing manuscripts, limited time, insufficient knowledge of publication standards, and insufficient research infrastructure [26]. Stone et al. also demonstrated the existence of institutional and non-institutional barriers to conducting research during undergraduate medical school. These barriers include time constraints, a lack of mentors, inadequate support, limited access to resources, curriculum design, a lack of skills and self-efficacy, awareness and motivation, funding, internet access, and gender and cultural issues, all of which hinder medical students’ engagement in research activities [9]. Furthermore, in prior studies, the unequal attainment gap among ethnic groups begged serious concerns about performance differences, therefore affecting medical education and the medical profession. The ethnicity of medical students often influences learning and performance due to limited educational resources, unadapted curricula, and medical school populations [27–29].

According to our knowledge, little is known about the practices, factors, and barriers affecting research engagement among medical graduates, especially when comparing national and international students. There are no other studies on medical undergraduate research in Romania, except for our previous study, which examined the first-time research perspectives and behaviors of students in their third and fifth years of study. The previous findings indicated that Romanian medical students value research possibilities, which promotes institutional attempts to support their curricular and extracurricular research [30]. This present study can be considered a continuation of the first investigation, as it aims to examine the factors that influence the engagement of undergraduate medical students in research, as well as the research practices performed by graduates until they complete their final year at the Faculty of Medicine of Iuliu Hatieganu University of Medicine and Pharmacy in Cluj-Napoca. This is one of the most prestigious medical universities in Romania. The university’s Faculty of Medicine admits three cohorts per year, and there are programs offered in various languages: Romanian, English, and French. The student selection process varies between programs. The Romanian program selects students for admission through a written exam. International applicants to the English and French language programs are admitted based on their academic performance and personal accomplishments. Although they share clinical areas and classrooms, local and foreign students do not show up to attend the same seminars. Every cohort has different clinical rotations and class schedules, so their academic activities never cross. Each year, the university’s Faculty of Medicine admits a specific number of students into the medical program. For example, in the last ten years, the admitted number of students per year varied between 500 and 600 students per year, until recent 4 years, when the university admitted approximately 800 students per year into its medical programs. The proportion of students has an equal distribution of 50% Romanian students and 50% international students [31]. The Cluj-Napoca Faculty of Medicine offers six-year undergraduate medical education that includes, in the first year’s curricula, a module on medical biostatistics and, in the second year’s curricula, a module on scientific research methodology. Until the final year, the students must prepare and present a demanding scientific report known as a graduation research thesis in accordance with the Bologna process. Teachers also offer guidance and support throughout extracurricular research.

This study aims to investigate the factors that encourage student engagement in research, as well as the barriers that limit their decision to participate in research. Furthermore, in terms of practices, behaviors for both mandatory and optional research activities have been followed. Furthermore, socio-demographic aspects were examined. This research would be valuable in creating an overview of the research motivation, barriers, and best practices for fostering research involvement in the current situation, while there is a persistent pedlary for medical students to become physician-scientists in the context of the physician-scientist deficit worldwide. This research seeks to provide insights into the research culture, resources available, and levels of student involvement in a medical school, along with potential differences between Romanian and international students in three graduating cohorts (2021–2023). Furthermore, examining the trends across graduation years may shed light on how medical education and research opportunities are evolving. If we understand students’ perspectives, we may use evidence-based ways to increase medical students’ interest and ameliorate barriers in research to prepare the future generation of physician-scientists.

The current research aimed to use a survey with 5-point Likert scales and multiple-choice questions to evaluate factors influencing research involvement and scientific activities among graduates from 2021 to 2023, along with exploring their socio-demographic characteristics. This study provided a focused examination of the following research objectives:

- Identification of socio-demographic indices: gender, section, and year of faculty graduation.
- Evaluation of factors that encourage student participation in research activities: personal influence, community influence, educational influences, and financial influences.
- Evaluation of the barriers that limit medical students research participation: personal influence, educational influences, and financial influence.
- Identifying research behaviors: the year of debut, complexity of research activity, contributions, participation in scientific congresses, participation in the process of writing a scientific article, aspects of publishing graduation thesis data in a scientific journal, and interest in participating in research activities after graduation.
- Comparing factors for involvement in research and scientific activities between Romanian and international students and analyzing them throughout time from 2021 to 2023.

## Material and methods

### Study sample and data collection

This research is a component of a larger study centered around evaluating the engagement of medical students in research and voluntary activities. The project received ethical approval from the Ethics Commission of Iuliu Hatieganu University of Medicine and Pharmacy under Approval Number DEP27/03.11.2021.

A cross-sectional investigation was conducted among graduate students of the Faculty of Medicine at the Iuliu Hațieganu University of Medicine and Pharmacy in Cluj-Napoca, Romania. From 2021 to 2023, all graduate students from the Romanian and international sections of the faculty were asked to participate in the study by filling out an anonymous online questionnaire (a total of 1878 students were invited). We chose to investigate the Romanian and international cohorts separately in order to learn more about how their educational and cultural backgrounds influence their research attitudes and practices. We separately looked at these groups to identify their unique requirements and obstacles in order to create focused strategies to increase student research participation. The questionnaire was distributed using the Microsoft Teams platform, which is commonly used by all affiliated members of the University of Medicine and Pharmacy, Iuliu Hatieganu. The students received an invitation explaining that participation was voluntary, and they agreed to participate by filling out the questionnaire. Those who did not wish to participate did not complete the questionnaire.

### Instrument for data collection

For this research project, we specifically designed an online survey to evaluate socio-demographic factors (age, gender), academic aspects (section, year of graduation), opinions about factors that encourage or limit involvement in research, and the research practices of undergraduate medical students. To identify common themes and factors reported in previous studies, we conducted a thorough literature review, which helped us derive the motivating factors and barriers related to student involvement in research. This influenced the development of our survey questions. Factors that encourage medical students involvement in research are the following: personal influence (motivation and personal interest, curriculum vitae improvement motivation), community influences (example of other colleagues), educational influences (teacher presentation of research participation options, teacher mentoring and support, medical research student courses or training), and financial influence (the existence of research grants for undergraduate students, monetary remuneration); The response choices were presented on a five-point scale that varied from “not at all” to “to a very high extent.” The barriers to medical students’ involvement in research are as follows: personal influence (lack of time caused by required medical training courses or internships during medical studies, lack of interest or lack of motivation for research), educational influences (difficulty finding a research coordinator, team, or research project), and financial influence (lack of or insufficient financial compensation for work done). The response choices were presented on a five-point Likert scale that varied from “not at all” to “to a very high extent.” Additionally, the questionnaire examined the research practices of medical students as follows: the year of study when students started their research activity, if they had been engaged in research projects only for their graduation thesis, or if they performed more complex research activities till graduation. The questionnaire asked about the contributions of students to research activities (data review of scientific literature, development of research ideas and hypotheses, research methodology and protocol, data gathering tools, statistical analysis, laboratory experiments, abstract and presentation development for scientific conferences, and writing medical articles). Moreover, the questionnaire asked about students’ involvement in medical congresses, if they had presentations such as oral or poster presentations (the response choices were presented on a four-point scale that varied from “not at all” to “more than three times”), if they had been involved in writing scientific articles (the response choices were presented on a four-point scale that varied from “not at all” to “more than three times”), or if they were publishing various types of scientific articles (publishing editorials or letters to the editor, reviews, original articles, clinical case presentations), and if they were first authors or co-authors. The students were asked if they had participated in research projects during medical school (the response choices were presented on a four-point scale that varied from “not at all” to “more than three times”). Additionally, the questionnaire asked about the interest in publishing graduation thesis data in a scientific publication. The questionnaire also evaluated interest in enhancing knowledge of proper scientific article writing, interest in better comprehension of abstract writing, and interest in understanding the publishing rules of a scientific paper. The questionnaire aimed to gather data on motivation and interest to participate in research activities after completing medical studies (with response options being ‘Yes,’ ‘No,’ or ‘I do not know’). Students received the questionnaire in Romanian, English, and French, and the average time to complete it was 15–20 minutes. We assessed the reliability of the questionnaire using internal consistency and found Cronbach’s alpha for each index. We found that the Research Involvement Index, which included 6 items, had a Cronbach’s alpha of 0.74; the Index of Factors Encouraging Student Research, which included 9 items, had a Cronbach’s alpha of 0.71; and the Research Involvement Barriers Index, which included 5 items, had a Cronbach’s alpha of 0.70. Each of the three indexes indicates good internal consistency. Our previous study, which examined the perspectives and behaviors of medical students in their third and fifth years of study for the first time, also tested the questionnaire. We made minor revisions to align with the actual research questions, thereby enhancing the questionnaire’s comprehensibility and reliability.

### Data analyses

The prevalence and mean values were calculated for the investigated topics separately for the Romanian section and international section, as well as for graduates from the 2021, 2022, and 2023 generations. Chi2 tests and t-tests were used to analyze differences among students in the Romanian and International sections, as well as among graduates from the 2021, 2022, and 2023 generations. Three types of indexes were developed to provide greater clarity into the factors influencing involvement in research and research practices.

An index of encouraging student research factors was developed by summing the scores (to a very high extent, coded + 2, to a high extent, coded + 1, I do not know, coded 0, to a low extent, coded − 1, not at all, coded − 2) of the following criteria: motivation and personal interest, curriculum vitae improvement motivation, examples of other colleagues, teacher presentations of research participation options, teacher mentoring and support, medical research student courses or training, the existence of research grants for undergraduate students, and monetary remuneration. The minimum value was − 16, and the maximum was + 16.

An index of research involvement barriers was developed by summing the scores (to a very high extent, coded + 2, to a high extent, coded + 1, I do not know, coded 0, to a low extent, coded − 1, not at all, coded − 2) of the following criteria: lack of time caused by required medical training courses or internships during medical studies, lack of interest or lack of motivation for research, difficulty finding a research coordinator, team, or research project, and lack of or insufficient financial compensation for work done. The minimum value was − 8, and the maximum was + 8.

An index for the involvement of medical students in research (research involvement index) was developed by summing the scores of involvements in the following research activities: participation at medical congresses, presenting papers at medical congresses (oral or poster presentations), participation in writing a scientific article, article publications, and participation in research projects. The available responses for each issue are 0 (no) and 1 (yes); therefore, the minimum value obtained for each participant was 0 and the maximum value obtained was 5.

We used forward selection in two stepwise multivariate linear regression analyses to find out what factors influenced the variations in the Research Involvement Barriers Index and the Index of factors that encourage student research. The dependent variables were the index of factors that encourage student research and the research involvement barriers index. For both, the independent variables were age, gender (coded 1–males, 2–females), and sections (Romanian section, international section). The analyses were performed separately for each index. Another stepwise multivariate linear regression analysis was conducted using forward selection to determine factors that contributed to the variation in the research involvement index. The dependent variables were the research involvement index, and the independent variables were age, gender (coded 1–males, 2–females), sections (Romanian section, international section), the index of factors that encourage student research, and the Research Involvement Barriers Index.

The data were analyzed using SPSS 22 statistical software, and significant findings are presented at a significance level of 0.05.

## Results

### Sociodemographic characteristics

The final sample included 572 participants, which represents a response rate of around 30%. Of the participants, 215 (37.6%) were male and 357 (62.4%) were female, aged between 22 and 54 years (mean 25.25, SD 2.1). Ranking them according to the study section, 392 (68.5%) were students from the Romanian section and 180 (31.5%) were students from the international sections. Ranking them according to the years of graduation, 232 (40.5%) students graduated in 2021, 172 (30%) in 2022, and 168 (29.5%) in 2023.

### Opinions on research and comparative analysis of graduate students from Romanian and international sections of different generations

Both Romanian and international students emphasize motivation, personal interest, and teacher mentoring and support as significant factors in research participation. Romanian students, in proportion to 67%, value motivation and personal interest, and 59% value teacher mentoring, while international students, in proportion to 58%, value motivation and personal interest, and 47% value teacher mentoring. Over one-third of Romanian students highlight CV improvement, research opportunities presented by teachers, and research training. Also, among international students, 40% report research training as influential, with around one-third citing CV improvement, examples of colleagues, and student research grants. The major barriers identified by Romanian students are as follows: 53% mention a lack of time and difficulty finding a research coordinator; 41% mention a lack of interest or motivation; and 20% mention insufficient financial compensation. Regarding the international students, 63% report difficulty finding a research coordinator, and 56% cite a lack of time, with a considerable proportion also noting financial constraints. The index of factors encouraging student research shows that Romanian students have a calculated score that varies between − 14 and + 16, with a mean of 8.38, whereas international students have a score ranging from − 4 to + 16, with a mean of 7.98. No statistically significant difference was seen between the two groups. The research involvement barriers index scores for Romanian students vary between − 6 and + 8, with a mean of 3.43, and for international students, they vary from − 4 to + 8, with a mean of 4.11. No statistically significant difference was seen between the two groups. Table 1 reports detailed information about the factors and barriers that could affect Romanian and international students’ participation in research activities.

**Table 1:** Research opinions - an analysis between Romanian and international students

Section	Romanian students	International Students
Factors that encourage student participation in research activities		
**Personal influence**		
** Motivation and personal interes**		
To very high extent (%) ^a^	66.3	58.9
To high extent (%) ^b^	29.8	37.2
I do not know (%) ^c^	0.8	0.6
To low extent (%) ^d^	3.1	2.8
Not at all (%) ^e^	0	0.6
Mean	1.59	1.51
**CV improvement motivation**		
To very high extent (%) ^a^	38.5	36.1
To high extent (%) ^b^	39	45.6
I do not know (%) ^c^	1.8	2.8
To low extent (%) ^d^	17.9	11.7
Not at all (%) ^e^	2.8	3.9
Mean	0.92	0 0.98
**Community influence**		
**Example of other colleagues**		
To very high extent (%) ^a^	24.7	28.9
To high extent (%) ^b^	38.5	45.6
I do not know (%) ^c^	1.8	2.8
To low extent (%) ^d^	29.1	19.4
Not at all (%) ^e^	5.9	3.3
Mean	0.47 *	**0.77**
**Educational influences**		
**Teacher presentation of research participation options**		
To very high extent (%) ^a^	43.9	35.6
To high extent (%) ^b^	44.9	49.4
I do not know (%) ^c^	0.3	1.1
To low extent (%) ^d^	9.7	11.1
Not at all (%) ^e^	1.3	2.8
Mean	1.20	1.03
** Teacher mentoring and support**		
To very high extent (%) ^a^	53.8	47.2
To high extent (%) ^b^	37.2	37.8
I do not know (%) ^c^	1	1.1
To low extent (%) ^d^	5.6	11.1
Not at all (%) ^e^	2.3	2.8
Mean	1.34 *	**1.15**
** Medical research student courses/training**		
To very high extent (%) ^a^	43.1	40.6
To high extent (%) ^b^	41.8	46.7
I do not know (%) ^c^	0.5	1.7
To low extent (%) ^d^	12	10
Not at all (%) ^e^	2.6	1.1
Mean	1.10	1.15
**Financial influence**		
**Student research grants**		
To very high extent (%) ^a^	43.9	36.1
To high extent (%) ^b^	45.2	37.8
I do not know (%) ^c^	0.3	5.6
To low extent (%) ^d^	9.2	15.6
Not at all (%) ^e^	1.5	5
Mean	1.20 **	**0.84**
** Monetary remuneration**		
To very high extent (%) ^a^	31.9	25
To high extent (%) ^b^	32.7	39.4
I do not know (%) ^c^	1	7.2
To low extent (%) ^d^	25.3	20
Not at all (%) ^e^	9.2	8.3
Mean	0.52	0.52
** Index of factors that encourage student research**		
Mean	8.38	7.98
Scor minim/ maxim	-14/+16	-4/+16
**Barriers to medical students’ research participation**		
**Personal influence**		
**Lack of time caused by required medical training courses/internships during medical studies**		
To very high extent (%) ^a^	52.3	63.3
To high extent (%) ^b^	31.9	26.1
I do not know (%) ^c^	0.8	0.6
To low extent (%) ^d^	12.2	8.3
Not at all (%) ^e^	2.8	1.7
Mean	1.18 *	1.41
**Lack of interest/motivation for research**		
To very high extent (%) ^a^	41.3	36.7
To high extent (%) ^b^	32.9	32.2
I do not know (%) ^c^	1	3.3
To low extent (%) ^d^	19.1	21.1
Not at all (%) ^e^	5.6	6.7
Mean	0.85	0.71
**Educational influences**		
**Difficulty finding a research coordinator/team/research project**		
To very high extent (%) ^a^	53.1	56.7
To high extent (%) ^b^	32.4	28.3
I do not know (%) ^c^	1.3	0.6
To low extent (%) ^d^	11.7	12.2
Not at all (%) ^e^	1.5	2.2
Mean	1.24	1.25
** Financial influence**		
Lack of/ insufficient financial compensation for work done		
To very high extent (%) ^a^	19.6	34.4
To high extent (%) ^b^	31.6	32.8
I do not know (%) ^c^	3.3	7.8
To low extent (%) ^d^	34.9	22.2
Not at all (%) ^e^	10.5	2.8
Mean	0.15 **	0.73
** Research involvement barriers index**		
Mean	3.43	4.11
Scor minim/ maxim	-6 /+8	-4/+8

*p* < 0.05- coded *, *p* < 0.01- coded **—statistically significant differences at t-test between Romanian and international medical students. ^a^- coded + 2, ^b^-coded + 1, ^c^-coded 0, ^d^-coded − 1, ^e^-coded − 2

Analyzing the answers of all students in the three graduating cohorts, several key factors emerged as influencing their involvement in research activities. The students consistently identified motivation, personal interest, teacher mentoring, and support as significant factors. Between 60% and 67% of all graduates attributed high importance to these factors. Teaching staff’s presentations of research opportunities, CV improvement, and the availability of student research funds enhanced the interest of about 40% of all cohorts of graduates in research. Colleagues’ examples and financial rewards significantly influenced the engagement of about 30% of 2023 graduates and one-third of 2021 and 2022 graduates. Throughout the years, barriers to research involvement remained consistent. Around half of students in all graduating cohorts identified a lack of time and difficulty finding a research coordinator, team, or project as major obstacles. Around 40% of graduates reported a lack of interest or motivation. Between 25% and 33% of graduates identified insufficient financial compensation as a significant barrier. However, the 2023 graduates placed more importance on the influence of examples from colleagues compared to the 2022 graduates. Furthermore, 2022 graduates emphasized the lack of funds as a barrier in comparison to 2021 graduates. The index of factors encouraging student research showed mean scores of 8.45 for 2021 graduates, 7.69 for 2022 graduates, and 8.57 for 2023 graduates, with no statistically significant differences between the groups.

The index of factors encouraging student research shows that 2021 graduates scored between − 7 and + 16, with a mean of 8.45. In comparison, 2022 graduates scored between − 14 and + 16, with a mean score of 7.69, while 2023 graduates scored between − 8 and + 16, with a mean score of 8.57. There was no statistically significant difference observed between the two groups. The Research Involvement Barriers Index scores for 2021 graduates range from − 6 to + 8, with a mean of 3.44; for 2022 graduates, the scores range from − 4 to + 8, with a mean of 3.78; and for 2023 graduates, the scores vary from − 3 to + 8, with a mean of 3.77. There was no statistically significant difference observed between the groups. Table 2 provides detailed information about the factors and barriers that could affect the students’ participation in research activities in the three graduating cohorts (2021–2023).

**Table 2:** Research opinions - an analysis comparing graduate students from the years 2021, 2022, and 2023

**Years**	**2021**	**2022**	**2023**
**Factors that encourage student participation in research activities**			
**Personal influence**			
**Motivation and personal interes**			
To very high extent (%) ^a^	67.2	61	62.5
To high extent (%) ^b^	28.9	34.3	34.5
I do not know (%) ^c^	1.3	0.6	0
To low extent (%) ^d^	2.6	3.5	3
Not at all (%) ^e^	0	0.6	0
Mean	1.60	1.51	1.56
**CV improvement motivation**			
To very high extent (%) ^a^	41.8	33.1	36.9
To high extent (%) ^b^	38.8	45.9	39.3
I do not know (%) ^c^	2.6	1.7	1.8
To low extent (%) ^d^	15.1	15.1	17.9
Not at all (%) ^e^	1.7	4.1	4.2
Mean	1.03	0.88	0.86
**Community influence**			
**Example of other colleagues**			
To very high extent (%) ^a^	29.7	18.6	28.6
To high extent (%) ^b^	36.6	43.6	43.5
I do not know (%) ^c^	1.7	2.9	1.8
To low extent (%) ^d^	28.9	26.7	21.4
Not at all (%) ^e^	3	8.1	4.8
Mean	0.61	**0.37**	**0.69**^t−b^*
**Educational influences**			
**Teacher presentation of research participation options**			
To very high extent (%) ^a^	43.1	38.4	41.7
To high extent (%) ^b^	45.3	49.4	44.6
I do not know (%) ^c^	0.4	0	1.2
To low extent (%) ^d^	9.9	9.3	11.3
Not at all (%) ^e^	1.3	2.9	1.2
Mean	1.18	1.11	1.14
**Teacher mentoring and support**			
To very high extent (%) ^a^	50.4	46.5	58.9
To high extent (%) ^b^	39.2	41.9	30.4
I do not know (%) ^c^	1.3	0.6	1.2
To low extent (%) ^d^	7.3	6.4	8.3
Not at all (%) ^e^	1.7	4.7	1.2
Mean	1.29	1.19	1.37
** Medical research student courses/training**			
To very high extent (%) ^a^	43.5	35.5	47.6
To high extent (%) ^b^	42.7	49.4	38.1
I do not know (%) ^c^	0.4	0.6	1.8
To low extent (%) ^d^	10.8	12.8	10.7
Not at all (%) ^e^	2.6	1.7	1.8
Mean	1.13	1.04	1.19
**Financial influence**			
**Student research grants**			
To very high extent (%) ^a^	43.5	41.3	38.7
To high extent (%) ^b^	40.9	42.4	45.8
I do not know (%) ^c^	1.3	2.3	2.4
To low extent (%) ^d^	10.8	11.6	11.3
Not at all (%) ^e^	3.4	2.3	1.8
Mean	1.10	1.08	1.08
**Monetary remuneration**			
To very high extent (%) ^a^	27.6	30.2	32.1
To high extent (%) ^b^	35.3	33.1	35.7
I do not know (%) ^c^	3.9	2.3	2.4
To low extent (%) ^d^	22.8	23.3	25
Not at all (%) ^e^	10.3	11	4.8
Mean	0.46	0.48	0.65
**Index of factors that encourage student research**			
Mean	8.45	7.69	8.57
Scor minim/ maxim	-7/+16	-14/+16	-8/+16
**Barriers to medical students’ research participation**			
**Personal influence**			
**Lack of time caused by required medical training courses/internships during medical studies**			
To very high extent (%) ^a^	55.2	55.8	56.5
To high extent (%) ^b^	28.4	30.8	31.5
I do not know (%) ^c^	0.4	1.2	0.6
To low extent (%) ^d^	12.1	10.5	10.1
Not at all (%) ^e^	3.9	1.7	1.2
Mean	1.18	1.28	1.32
**Lack of interest/motivation for research**			
To very high extent (%) ^a^	40.1	40.1	39.3
To high extent (%) ^b^	32.3	33.7	32.1
I do not know (%) ^c^	1.3	2.3	1.8
To low extent (%) ^d^	21.1	18	19.6
Not at all (%) ^e^	5.2	5.8	7.1
Mean	0.81	0.84	0.76
**Educational influences**			
Difficulty finding a research coordinator/team/research project			
To very high extent (%) ^a^	57.8	48.8	54.8
To high extent (%) ^b^	26.3	35.5	33.3
I do not know (%) ^c^	1.3	1.2	0.6
To low extent (%) ^d^	12.5	13.4	9.5
Not at all (%) ^e^	2.2	1.2	1.8
Mean	1.25	1.17	1.30
**Financial influence**			
Lack of/ insufficient financial compensation for work done			
To very high extent (%) ^a^	22	27.3	24.4
To high extent (%) ^b^	28.9	34.3	33.9
I do not know (%) ^c^	6	4.7	3
To low extent (%) ^d^	32.3	26.7	33.3
Not at all (%) ^e^	10.8	7	5.4
Mean	0.18^t−a^*	0.48	0.38
**Research involvement barriers index**			
Mean	3.44	3.78	3.77
Scor minim/ maxim	-6/+8	-4/+8	-3/+8

*p* < 0.05- coded ^t-a*^—statistically significant differences at t-test between 2021–2022 graduate medical students

*p* < 0.05- coded ^t-b*^—statistically significant differences at t-test between 2022–2023 graduate medical students

^a^- coded + 2, ^b^-coded + 1, ^c^-coded 0, ^d^-coded − 1, ^e^-coded − 2

### Practicies on research and comparative analysis of graduate students from Romanian and international sections of different generations

Around one-third of students from both sections began participating in research during their sixth year, with Romanian students starting earlier on average (t-test, *p* < 0.01). About 70% of Romanian and over 80% of international students engaged in research linked to their graduation thesis, with a significant difference between groups (chi-square, *p* < 0.05). Less than 20% performed more complex research. Romanian students more frequently participated in data collection compared to international students who preferred performing literature reviews (chi-square, *p* < 0.01). Around 80% of Romanian and less than half of international students attended medical conferences (chi-square, *p* < 0.01). In proportion, 36% of Romanian and 21% of international students were on the scientific meetings organization staff (chi-square, *p* < 0.01). Approximately 12% of Romanian and 5% of international students presented posters at scientific conferences (t-test, *p* < 0.05). One-quarter of Romanian and 20% of international students contributed to the writing of medical research papers, with Romanian students having a higher co-authoring rate (chi-square, *p* < 0.05). A proportion of 29% of Romanian and 20% of international students were interested in publishing their research data (chi-square, *p* < 0.05). Overall, 7% of international students and 6% of Romanian students have published their graduation thesis output. The research engagement index was higher for Romanian students (mean 1.53) compared to international students (mean 1.06) (t-test, *p* < 0.01). Over 80% of students showed interest in improving their skills in scientific writing, with higher interest among Romanian students (chi-square, *p* < 0.05), and around 60% were interested in post-graduation research activities. Table 3 provides detailed information about research practices and comparative analyses of Romanian and international graduates.

**Table 3:** Research practicies- An analysis between Romanian and international students

Section	Romanian students	International Students
Student research participation		
Commencing in Year I (%)	1.5	1.1
Commencing in Year II (%)	7.1	2.2
Commencing in Year III (%)	3.3	2.2
Commencing in Year IV (%)	6.1	6.7
Commencing in Year V (%)	13.5	30.6
Commencing in Year VI (%)	29.3	33.9
Not at all	39	23.3
Mean	**2.94**^t^**	**3.95**
**Research was exclusively linked with graduation thesis (%)**	70.7*	79.4
**The research work was more complex (%)**	17.1	12.2
**Lack of research (%)**	12.2	8.3
**Contributions to research activities**		
**Data review of the scientific literature (%)**	32.4**	44.4
**Development of research ideas/hypotheses (%)**	28.8	22.8
**Development of the research methodology and protocol (%)**	25.5	20
**Development tools for gathering data (%)**	22.2	17.2
**Data gathering in many contexts such as communities, hospitals, or other organizations (%)**	35.5**	24.4
**Data statistical analysis (%)**	37.2	37.2
**Performing laboratory experiments (%)**	9.2	10
**Creating abstracts and presentations for scientific conferences (%)**	12	9.4
**Writing medical articles (%)**	14	10
**Graduation thesis writing (%)**	69.9	66.7
**No involvement in research projects (%)**	14.3	9.4
**Attending medical conferences without presenting a scientific paper (%)**	80.1**	46.1
**Organizing student scientific meetings or events (%)**	36.5**	21.1
**Scientific paper presentations at scientific congresses**		
Once (%) ^a^	7.7	6.6
Two to three times (%) ^b^	4.8	2.8
More than three times (%) ^c^	2	1.1
Not at all (%) ^d^	85.5	89.4
Mean	0.23	0.15
**Presentation of posters at scientific conferences (%)**	11.7*	5
**Oral presentation of scientific findings at scientific congresses (%)**	7.1	10
**Presenting posters and performing oral presentations at scientific conferences (%)**	4.3	4.4
**Winning congresses awards (%)**	2.6	2.2
**Participating in the writing process of a scientific article**		
Once (%) ^a^	15.1	12.8
Two to three times (%) ^b^	7.1	5
More than three times (%) ^c^	2.6	1.7
Not at all (%) ^d^	75.3	80.6
Mean	0.36	0.27
**Published article (%)**	14.5	9.44
**Article published as first author (%)**	3.6	5
**Article published as co-author (%)**	12.5*	6.1
**Publishing Editorial/Letter to the Editor (%)**	0	0
**Publishing review (%)**	5.4	7.8
**Publication Original Article (%)**	7.9	6.1
**Clinical Case Presentation Publication (%)**	7.1	5
**Concerns about publishing graduation thesis data in a scientific journal**		
**Students interested in disseminating data (%)**	29.3*	20.6
**Article in peer review for a medical journal (%)**	0**	3.3
**Article accepted for publication in a scientific journal, but not yet published (%)**	2.3	2.2
**Data is published in a scientific journal (%)**	3.8	4.4
**Disinterested in data publication (%)**	64.5	67.7
**Participation in a research project during medical studies**		
Once (%) ^a^	14.5	16.1
Two to three times (%) ^b^	3.6	3.9
More than three times (%) ^c^	1	0.6
Not at all (%) ^d^	80.9	79.4
Mean	0.24	0.25
**Earning research projects (%)**	1.5	2.2
**Research involvement index**		
0 (%)	16.5	0
1 (%)	48.4	41.1
2 (%)	18.1	38.3
3 (%)	13.5	13.8
4 (%)	3.3	5.5
5(%)	0	1.1
Mean	1.53^t^**	1.06
**Students interested in improving their knowledge of the correct writing of a scientific article (%)**	88.5*	81.7
**Students interested in improving their comprehension of abstract writing (%)**	87.5**	78.3
**Students interested in improving their understanding about the publication rules of a scientific article (%)**	89.5*	82.2
**Students who wish to engage in research activities after finishing their education (%)**		
Yes (%) ^e^	61.5	58.9
I don’t know (%) ^f^	29.8	41.1
No (%) ^g^	8.7	0
Mean	0.61	0.58

*p* < 0.05- coded *, *p* < 0.01- coded **—statistically significant differences at chi 2 test between Romanian and international medical students

*p* < 0.05- coded ^t^*, *p* < 0.01- coded ^t^**—statistically significant differences at t-test between Romanian and international medical students

^a^-coded 1, ^b^-coded 2, ^c^-coded 3, ^d^-coded 0, ^e^-coded + 1 ^f^-coded 0, ^g^-coded − 1

Approximately one-third of each cohort began research in their sixth year, with 2022 graduates starting earlier on average (t-test, *p* < 0.05). Over 70% of graduates from all years participated in thesis-linked research, while less than 20% conducted more complex research. Around 31–38% of participants reviewed scientific literature, 25% developed research ideas and methodologies, and 28–37% performed data collection. More than one-third of 2021 graduates, as well as 40% of 2022 and 2023 graduates, performed statistical analysis. Most students attended medical congresses, with 12–15% presenting papers, 9% presenting posters, and 6.5–9.9% giving oral presentations. A quarter of 2021 graduates, 42% of 2022 graduates, and 30% of 2023 graduates were on the scientific meetings organization staff, with higher engagement in 2022 and 2023 (chi-square, *p* < 0.05). Around 23% of graduates contributed to writing medical research papers. About 29% of 2021 graduates and 25% of 2022 and 2023 graduates were interested in publishing their research data, while 6% of the three graduating cohorts had accepted or published articles. Approximately 20% of graduates engaged in faculty research projects, with a mean of 1.3 regarding the research index scores. Interest in improving scientific writing skills was high. Over 79% of graduates showed interest in improving their skills in scientific writing, with higher interest among 2022 and 2023 graduates (chi-square, *p* < 0.05), and around 60% were interested in post-graduation research activities. Table 4 provides detailed information about practices in research and comparative analysis in the three graduating cohorts (2021–2023).

**Table 4:** Research practicies- An analysis comparing graduate students from the years 2021, 2022, and 2023

Years	2021	2022	2023
**Student research participation**			
Commencing in Year I (%)	1.3	1.2	1.8
Commencing in Year II (%)	3.9	7.6	6
Commencing in Year III (%)	3.4	2.9	2.4
Commencing in Year IV (%)	6.5	5.8	6.5
Commencing in Year V (%)	22.4	14.5	18.5
Commencing in Year VI (%)	31	27.9	33.3
Not at all	31.5	40.1	31.5
Mean	**3.44**^t−a^*	**2.88**	3.39
** Research was exclusively linked with graduation thesis (%)**	71.6	74.4	75
** The research work was more complex (%)**	18.1	15.7	11.9
** Lack of research (%)**	10.3	9.9	13.1
**Contributions to research activities**			
**Data review of the scientific literature (%)**	38.8	37.8	31
**Development of research ideas/hypotheses (%)**	24.6	28.5	28.6
**Development of the research methodology and protocol (%)**	22.8	26.2	22.6
**Development tools for gathering data (%)**	19.4	18	25
**Data gathering in many contexts such as communities, hospitals, or other organizations (%)**	28.4	32	36.9
**Data statistical analysis (%)**	34.9	37.8	39.9
** Performing laboratory experiments (%)**	8.6	11.6	8.3
** Creating abstracts and presentations for scientific conferences (%)**	8.6	14	11.99
** Writing medical articles (%)**	13.4	14	10.7
** Graduation thesis writing (%)**	65.5	74.4	67.9
** No involvement in research projects (%)**	13.8	9.9	14.3
** Attending medical conferences without presenting a scientific paper (%)**	72	66.3	69
** Organizing student scientific meetings or events (%)**	25^a^**	42.4	29.8^b^*
**Scientific paper presentations at scientific congresses**			
Once (%) ^a^	7.75	8.72	5.35
Two to three times (%) ^b^	4.31	4.65	3.57
More than three times (%) ^c^	0.86	1.74	3.37
Not at all (%) ^d^	87	84.8	88
Mean	0.18	0.23	0.21
** Presentation of posters at scientific conferences (%)**	9.9	9.3	9.5
** Oral presentation of scientific findings at scientific congresses (%)**	7.8	9.9	6.5
** Presenting posters and performing oral presentations at scientific conferences (%)**	4.7	4.1	4.2
** Winning congresses awards (%)**	2.2	2.9	2.4
**Participating in the writing process of a scientific article**			
Once (%) a	13.4	15.1	14.9
Two to three times (%) b	6.9	5.8	6.5
More than three times (%) c	2.6	2.3	1.8
Not at all (%) d	77.2	76.7	76.8
Mean (%)	0.34	0.33	0.33
** Published article (%)**	13.3	12.7	12.5
** Article published as first author (%)**	4.7	2.9	4.2
** Article published as co-author (%)**	9.5	11	11.3
** Publishing Editorial/Letter to the Editor (%)**	0	0	0
** Publishing review (%)**	6	5.2	7.1
** Publication Original Article (%)**	6.9	8.7	6.5
** Clinical Case Presentation Publication (%)**	5.6	6.4	7.7
**Concerns about publishing graduation thesis data in a scientific journal**			
** Students interested in disseminating data (%)**	28.9	24.4	25.6
** Article in peer review for a medical journal (%)**	**1.7**	1.2	0^b*^
** Article accepted for publication in a scientific journal, but not yet published (%)**	2.6	1.2	3
** Data is published in a scientific journal (%)**	3.9	4.7	3.6
** Disinterested in data publication (%)**	45.7	4.1	4.2
**Participation in a research project during medical studies**			
Once (%) a	15.9	14.5	14.3
Two to three times (%) b	2.2	3.5	6
More than three times (%) c	0.9	1.2	0.6
Not at all (%) d	81	80.8	79.2
Mean	0.22	0.25	0.27
**Earning research projects (%)**	1.3	1.7	2.4
**Research involvement index**			
0 (%)	9	16.2	9.5
1 (%)	46.1	43	49.4
2 (%)	27.5	20.9	23.8
3 (%)	12.9	16.2	11.9
4 (%)	3.4	3.4	5.3
5 (%)	0.8	0	0
Mean	1.40	1.37	1.38
** Students interested in improving their knowledge of the correct writing of a scientific article (%)**	81.5^a^*	90.1	89.3^b^*
** Students interested in improving their comprehension of abstract writing (%)**	79.7^a^*	88.4	87.5^b^*
** Students interested in improving their understanding about the publication rules of a scientific article (%)**	86.2	87.8	88.1
**Students who wish to engage in research activities after finishing their education (%)**			
Yes (%) ^e^	58.2	58.1	66.7
I don’t know (%) ^f^	37.5	41.9	33.3
No (%) ^g^	4.3	0	0
Mean	0.58	0.58	0.66

*p* < 0.05- coded a*, *p* < 0.01- coded a** —statistically significant differences at chi 2 test between 2021–2022 graduate medical students

*p* < 0.05- coded b*, *p* < 0.01- coded b** —statistically significant differences at chi 2 test between 2021–2023 graduate medical students

*p* < 0.05- coded t-a*, *p* < 0.01- coded t-a**—statistically significant differences at t-test between 2021–2022 graduate medical students

^a^-coded 1, ^b^-coded 2, ^c^-coded 3, ^d^-coded 0, ^e^-coded + 1 ^f^-coded 0, ^g^-coded − 1

Regarding aspects associated with involvement in research, the multivariate linear regression findings show that the index of positive factors was higher among female students (standardized beta 0.146, CI = 4.715–7.322, *P* < 0.01). Additionally, the negative factor index was shown to be higher among female students (standardized beta 0.144, CI = 0.363–1.308, *P* < 0.01) and in international sections (standardized beta 0.131, CI = 0.296–1.282, *P* < 0.01). Also, the research index was higher among the Romanian section (standardized beta − 0.174, CI = -0.688–-0.251, *P* < 0.01).

## Discussion

This study investigates the research factors and practices of students in their final year at Cluj-Napoca’s Iuliu Hatieganu University of Medicine and Pharmacy Faculty of Medicine.

The concept of originality is related to the evaluation of the aspects perceived by medical students regarding the factors that encouraged engagement in research activities, as well as the barriers that restricted their involvement, until the final year of study. It also refers to determining potential disparities in research culture and in student involvement in different types of research opportunities among Romanian and international medical graduates. Furthermore, performing an analysis of the patterns observed across different graduating years (2021–2023) may provide valuable insights into the dynamic nature of medical education and the potential for research advancements.

### Factors encouraging and maintaining interest in medical student research

Ommering et al. found that to encourage and maintain the interest of medical students in research, it is necessary to understand the motivations that drive them to engage in research as well as the specific factors that contribute to their motivation for research [23]. In this light, our study’s results indicate that personal interest, which represents intrinsic motivation, is the most important factor that significantly encourages student engagement in research. Additionally, the authors of the previous cited study found that students may undertake research for future educational and professional options, such as a desired residency position [23]. However, our study reveals that the improvement of the curriculum vitae, a representation of extrinsic motivation, appears to have a less significant impact on students’ involvement in research. It’s possible that the lower significance achieved by improving their CV is due to the fact that, in the Romanian medical system, training possibilities and jobs post-graduation are based primarily on exams rather than CVs [30]. The absence of observed discrepancies between both sections is intriguing because this aspect was anticipated to have a greater impact on students from the international sections as the curriculum vitae continues to have significant importance in the residency applicant assessment process for most graduates globally [32]. Thus, according to our findings, medical schools should prioritize their students’ personal interests and curiosity in research. This might entail both research classes and practical research activities as part of the teaching program, which should promote curiosity and foster intrinsic motivations early in medical education.

### **Institutional factors influencing research involvement**

In this study, educational influences, such as the presentation of research participation options by teachers, their mentoring and support, and the organization of medical research student courses or training, have a significant impact on students’ involvement in research. According to Abu-Zaid, teachers who encourage research have a substantial impact on students’ views towards this area and their aspirations for future careers [33]. However, the significance of teacher mentorship and assistance is perceived to a greater extent by students in the Romanian section. The observed disparity between the sections is unexpected, as both Romanian and international students interested in medical research receive the same guidance and assistance for research participation. This is due to the fact that the “Iuliu Hatieganu” University of Medicine and Pharmacy actively promotes research activities across all fields and departments. One potential reason for this disparity could be cultural differences in the perception of mentorship. Given their different origins, international graduates could have different expectations and mentorship experiences. Although the university strives to provide comparable mentoring, the increased perceived value of teacher interaction among Romanian students indicates underlying reasons needing further investigation.

Furthermore, when considering financial factors, it is observed that students view the presence of research grants as a significant and favorable factor that encourages their engagement in research. Similar findings were also expressed by Australian students, who said that one of the main elements motivating research activities throughout medical school is financing [34]. Iuliu Hatieganu University of Medicine and Pharmacy ranks first among Romanian medical universities in the number and value of competitive research grants due to the extraordinary effort of teaching staff collectives, the institutional frame improved by creating the Department for Research and Development, and the more generous financing programs. Most research funding comes from grants and contracts [35]. However, the results of the present investigation showed that Romanian students expressed a stronger belief that the existence of funds has a higher impact on their engagement in research. Romanian students probably view financing as more significant because of their connections with local funding sources, prior expertise in financially sponsored research projects, cultural and socioeconomic issues, and favorable experiences with financed research. To enhance research engagement, it should provide customized support and resources, encourage collaborative research efforts, and promote cross-cultural learning and idea exchange.

### Community influences

The benefits of collegiality and collaboration, knowledge acquisition, and career-mindedness for medical students were highlighted by Yin et al. in their investigation that examined the effects of graduates’ research experiences on their medical undergraduate colleagues. [16]. The current study found that the example of other colleagues influences their involvement in research, and the findings vary between the groups under investigation. International students place a higher importance on this factor, probably because they could be more collaborative with their colleagues in the context of their smaller number of colleagues than in the Romanian section. Thus, they could have more chances to work together on research projects and influence each other by personal example. Additionally, the cohort of 2023 graduates showed stronger confidence that the influence of their colleagues’ examples has a greater effect on their research engagement compared to the 2022 graduate cohort. This might be the result of more peer cooperation, more group research projects, or a developing university culture of common academic interests.

### Barriers to research participation

The outcomes of our study correspond closely to the available literature; many of the findings regarding barriers are comparable to the results of previous investigations. Key barriers to undergraduate research participation include a lack of knowledge and skills, limited faculty support and funding, as well as structural barriers like time constraints, limited research facilities, and a lack of motivation [36]. Our findings highlighted that the time constraints caused by time-consuming internships or mandatory medical training courses are the most significant obstacle impeding students’ engagement in research activities. According to our findings, “lack of time” has a greater impact on international students, who may have less time to do research because they must adapt to new educational systems and learn a new language. It is already known that medical curricula are often too rigorous to include sufficient time for extracurricular study [37]. Siemens et al. also identified a lack of time as a major obstacle to conducting research, citing a demanding school schedule [38]. Most students perceive the challenge of finding a research coordinator or team and a research project as a significant obstacle. Similar studies on the importance of research mentorship for medical students mirrored our findings [38, 39]. In addition, their lack of interest in research and lack of or insufficient financial remuneration are perceived as minor barriers by respondents. Hegde et al. and Kumar et al. also demonstrated similar results, describing barriers such as lack of interest, funding, and poor availability of research mentors that can hinder undergraduate participation in research [39, 40]. Developing flexible curricula, enhancing mentoring programs, developing research skills, offering time management support, and improving funding possibilities will help students participate in research without compromising their clinical training or academic responsibilities.

### Integration of research into medical curricula

The Boyer Commission’s report on undergraduate medical education emphasizes the importance of integrating scientific research training into medical curricula. This trend has evolved, and currently, research-based learning is widespread. Medical schools engage students in undergraduate research in various ways. Research-driven courses, extracurricular activities, and graduate research projects are examples [24, 41]. Medical students at Iuliu Hatieganu University of Medicine and Pharmacy Faculty of Medicine participate in both compulsory research and extracurricular activities. Table 5 summarizes the main activities. These activities should improve abilities in critical literature evaluation, study objectives, methodology, data collection, analysis, interpretation, and oral presentation [30]. Incorporating scientific research in medical education at an early stage improves both cognitive and practical abilities, develops intellectual skills, encourages evidence-based learning, promotes the production of publications, stimulates future research, and facilitates career progression [33]. Although there are different opinions about compulsory research in the faculty. According to Abu-Ziad et al., this could lead to bad research practices that harm universities and research organizations.

**Table 5:** Overview of Research activities and support for students

Category	Activity	Description
Required Research Activities	Medical Biostatistics and Informatics	Undergraduates are working with databases, performing statistical analyses, and interpreting and presenting results.
	Medical Research Methodology	Aims to enhance skills in retrieving, using, and evaluating medical scientific literature, selecting appropriate research methods, data analysis, interpreting results, presenting results, and practicing evidence-based medicine
	Graduation thesis	Conducting a comprehensive review of relevant academic literature. Gathering data through experiments, surveys, or other methods. Analyzing and interpreting collected data. Writing and presenting the final research thesis.
Optional Research Activities	Optional classes organized by Medical Informatics and Biostatistics department	“Poster, PowerPoint and Examination clinical case presentation - from theory to practice” “Short methodological guide for the diploma thesis” “Critical reading of medical articles in evidence-based medicine” “I want to publish”
	Conference Attendance	Attending academic conferences to present research findings.
	Medical Research education-student organization	The project involves organizing conferences, trainings, and workshops to teach volunteers research basics and involving students in research projects through academic staff collaborations.
	Scientific Circles	Engaging in research projects organized by the departments of the Faculty of Medicine with other students.
	Faculty Research Centres	Participation in research teams
Research Support Available	Faculty Mentorship	Guidance and support from faculty advisors throughout the research.
	The Department of Research, Development and Innovation	Financial support for research activities through grants and funds.
	Access to Laboratories and Equipment	• Biobase – Center for Experimental Medicine and Practical Skills. • Genomics Research Centre. • MedFUTURE Research Centre.
	Valeriu Bologa” Library	Access to academic journals, databases, and other research materials.

### Student involvement in research activities

The findings of the investigation indicated that most students commenced their research activities at a later stage, predominantly during their fifth and sixth years of study. This research commencement coincides with the most common timeframe for starting graduate research. Furthermore, the proportion of students who participated in more complex research activities varied from 12 to 18%. However, their research roles have been vast. These include a data review of scientific literature, the formulation of research ideas and hypotheses, the development of research methodology and protocol, the creation of data collection tools, data gathering in various environments, including hospitals, communities, and organizations, and data statistical analysis. The percentage of students engaged in activities such as conducting laboratory experiments, writing medical articles, and developing abstracts and presentations for scientific conferences was considerably lower. Romanian students were more involved in data collection, while international students focused more on literature reviews. The language barrier could be the key to these results, as international students could perform review-type research more easily than gathering data from local patients, while Romanian students were expected to collect information more easily due to their access to patient data and their improved interactions with local patients.

### Data dissemination

Romanian students and international students have significantly different participation rates in medical conferences. Events like conferences, workshops, seminars, and symposiums offer unique learning opportunities. These events encourage medical staff to remain current on research, discuss best practices, and learn new skills, developing safety and quality [42]. Romanian students have a higher percentage of presentations, with around 15% presenting their work, while international students have around 10%. Posters were more common among Romanian students, while oral presentations were more common among international students. Our findings align with a previous study conducted in the United Kingdom, which showed that 17% of students had submitted an article for scientific meetings, which refers to their participation in poster and podium presentations [26].

Between 20% and 25% of students from the studied groups have contributed to writing medical publications at least once, while between 10% and 15% of participants published papers as authors. The Romanian section had a higher percentage of students who co-authored papers. Students from both sections contributed reviews, original articles, and clinical case presentations. Similarly, a previous investigation conducted among students from Dutch universities showed that 12% of the participants had published one or more papers either prior to or during their year of graduation [43]. In their study, Barbosa et al. showed that investigations conducted at the medical-degree level are an unexplored resource of scientific knowledge. Active participation in scientific research holds significant value in terms of enhancing one’s personal knowledge. However, it is equally crucial to share this knowledge to advance the medical field and, subsequently, improve healthcare outcomes [8]. More than one-quarter of students expressed interest in publishing their graduation research data, with Romanian students showing more interest. This may be due to the fact that most international graduates do not continue their training in Romania after graduation, making it difficult to work with the research team to disseminate graduation study results. Currently, there are international students with at least one paper at the peer review stage. Also, under 10% of students have articles approved or published already. Therefore, the publication rate for research graduation theses was lower than that of other European studies, with rates of 10.4% in Portugal, 17% in France, and 23.8% in Finland [8]. To contrast, our study exposed data collected around graduation, while these studies revealed data collected years after graduation [8].

### Importance of research writing skills and career motivation

Previous investigations showed that medical students need expertise in writing papers and abstracts. Teaching these abilities would be valuable, and medical schools should provide information and knowledge about writing scientific articles and abstracts to help students develop a solid foundation for their postgraduate medical careers [26]. Our findings demonstrated that almost all the students want to improve their scientific manuscript writing (writing of the scientific article, abstract) and publishing guidelines. The 2022 and 2023 graduates were more interested in learning how to write a scientific article and abstract writing, while the Romanian students were more interested in improving their scientific manuscript writing and publishing guidelines.

According to Waaijer et al., positive experiences can drive student motivation in a research career. Thus, the present investigation showed that over half of participants express a desire to continue conducting research after graduation, and they are probably likely to have had favorable experiences related to research throughout their medical school studies [43]. Moreover, a systematic review focused on career choice demonstrated that obtaining a medical degree or participating in a fellowship program is linked to a professional path in the field of research medicine. Also, the completion of research projects and subsequent dissemination of findings within the context of medical school and residency have a strong connection to a career path in the field of research medicine [44].

### Strengths and limitations of the study

There are several limitations associated with this study. The first limitation could be the fact that the study provides valuable insights into research participation among Romanian and international medical graduates; the findings could be comparable only with those of other medical schools under the Bologna process that adopt similar curricular and extracurricular research activities. Furthermore, the research sample includes exclusively medical graduates from one Romanian medical institution, so the findings could restrict the representation of many points of view and experiences in the larger community of medical graduates. Moreover, participants who are more interested in research may self-select, which could influence the findings. Another possible limitation of our study is the low response rate observed. We also observed declining participation rates over successive years. Survey fatigue, demographic changes, methodologies, perceived relevance, privacy issues, benefits, and societal trends all could help to explain declining survey participation rates. Also, uncontrollable factors such as socioeconomic status, prior research experience, or personal motivations can complicate the relationship between identified variables and barriers to research participation, thereby complicating the ability to establish causal relationships. Moreover, the cross-sectional design of the study may restrict its ability to capture changes in research participation. It is very difficult to observe patterns and experiences over time or across different stages of medical education. However, a strong point of this study can be considered a continuation of the first investigation, as it aims to examine the factors that influence the engagement of undergraduate medical students in research in their third and fifth years of study, who graduated in 2021 and were part of the study’s sample.

## Conclusions

The findings of this study offer important perspectives into the involvement of medical undergraduates in research during medical school, as well as the factors and barriers that interfere with research participation. The results demonstrate that intrinsic motivation is the primary factor driving student engagement in research, while institutional factors, such as educational, financial, and community influences, also have a substantial impact on research involvement. Lack of interest and time restrictions are the two main barriers. Furthermore, observed were financial issues, difficulties finding a research coordinator or team, and securing a research project. Also, this study revealed the existence of research culture differences between Romanian and international students and underlined the dynamic character of medical education. This work could be used as a foundation for future research to explore methods for removing these obstacles and fostering factors that may impact research engagement. These results could be adapted by teaching staff about practical medical education to offer effective strategies for encouraging undergraduate research field involvement and promoting cross-cultural learning. Also, universities and policymakers could utilize these findings to concentrate their initiatives on reducing the main barriers to achieving high-quality research. Overall, this study not only advances academic understanding but also offers tangible benefits to all parties involved, fostering a collaborative approach to encourage research participation among medical undergraduates.

## Data Availability

The datasets utilized and analyzed in the present study are accesible upon resonable request from the corresponding author.

## Declarations

The need for written informed consent was waived by the Iuliu Hatieganu University of Medicine and Pharmacy Ethics Committee due to the nature of the survey, which did not collect any personally identifiable data.

All students were informed that participating in the study is voluntary and that they can choose not to participate by not completing the anonymous survey. The students who completed the survey expressed their agreement with their involvement in the study.
